# Psychiatric Inpatient Services in a General Hospital Setting in the City State of Singapore: An Attempt to Improve the Inpatient Experience of Patients with Multi-Disciplinary Approach

**DOI:** 10.1192/bjo.2025.10387

**Published:** 2025-06-20

**Authors:** Palanivelu Sendhil Kumar, Chao Tian Tang, Ho Teck Tan, Kar Yin Lee, Su Yin Seow

**Affiliations:** Sengkang General Hospital, Singapore, Singapore

## Abstract

**Aims:** Sengkang General Hospital (SGH) is one of the newest government hospitals in the city state of Singapore. This busy 1000 bedded hospital has a 14 bedded psychiatric unit managed by the Department of Psychiatry. The department has 9 specialists, 4 to 5 Junior doctors and is supported by Allied Health Professionals like Psychologists, Occupational Therapists, Medical Social Workers and Nursing Professionals. All the above-named professionals work together as a well-oiled unit caring for the mental health needs of the patients mainly in the North East of Singapore. Ward 45 in SGH where the psychiatric unit is located houses inpatients who are 18 years and above. The patients have varied diagnosis, present with different risk profiles and as expected in any inpatient unit have varied lengths of stay. The aim was to start an inpatient programme which would benefit the patients during their stay and help in their recovery as well as equip them to build on their recovery and get back to successfully living in the community.

**Methods:** At the outset a few sessions were arranged involving all the professionals to discuss the therapeutic needs of the inpatients and how they could be addressed. The main aim of the programme was to help in recovery and relapse prevention. A list was compiled and in subsequent sessions the different health professionals who could deliver that was then mapped out. Once this was clear the individuals took it back to their respective departments to finalise on the deliverables and scheduling. This whole process took about a month. Once we were clear on the individual roles, we submitted the manpower requirement and time requirement for the Hospital Finance to generate a service code based on which the charges could be implemented. In subsequent meetings with all the professionals the different dates on which each professional could deliver the inpatient activity was finalised. The programme went live in February 2024. After a 3 month period feedback was obtained from patients and also the professional;s involved and some minor changes were made. We have now completed a year of the programme. A sample of type of activity is given below:

Psychology:

My distraction plan (pleasurable activities + thinking of someone patient cares about).

Self-soothing plan (self-compassion).

Un/helpful thinking styles.

Sleep hygiene + plan.

Values + action plan.

Self-esteem (kindness meditation).

Coping statements.

Relaxation (deep breathing + PMR).

Occupational Therapist:

Painting.

Collage.

Craft.

Drawing.

Medical Social Worker:

Generic activities to improve interpersonal skills and functioning.

Therapeutic approaches like IPT and DBT.

Nursing:

Psychoeducation activities about their condition.

Psychoeducation around the medications the patient is on.

Personal care advice and training.

**Results:** The Programme was accepted and appreciated by majority of the patients. The healthcare professionals also enjoyed delivering various therapeutic aspects to the patients and took an active role to improve care of the patients. A survey was done which captures the patients’ views on some aspects of the programme by different professionals. The results have been overwhelming and a high percentage of patients have rated the programme as appropriate, useful and recommendable to others. A brief tabulation of the survey has been posted below:

Psychology: 76% of the participants found the programme useful in their treatment journey; 73% of the participants would recommend the programme to other service users.

Occupational Therapist: 89% of the participants found the programme useful in their treatment journey; 93% of the participants would recommend the programme to other service users.

Medical Social Workers: 75% of the participants found the programme useful in their treatment journey; 75% of the participants would recommend the programme to other service users.

Nursing: 63% of the participants found the programme useful in their treatment journey; 69% of the participants would recommend the programme to other service users.

**Conclusion:** The response to the initiative and encouragement has been overwhelming. We would like to enhance and build on the progress. Another regional hospital in Singapore has expressed interest in learning from our model and we intend to assist them in any way and collaborate and build on what we have gained. We are in the process of collecting data to see if we have made any progress on relapse prevention. Some of the things we can improve on:

The duration of the sessions as some patients stay short periods hence miss out.

Some patients who are high risk are excluded and we need to devise ways to include them.

Increasing awareness and also devise means of reducing the financial burden for participation in the programme.

Constantly look at what is delivered and how a varied and broad category of interventions can be provided (prevent repetition).

